# The Practical Treatment of Stammering and Stuttering, with Suggestions for Practical and Helpful Exercises

**Published:** 1903-10

**Authors:** 


The Practical Treatment of Stammering and Stuttering, With Suggestions for Practical and Helpful Exercises.—By George Andrew Lewis; and a Treatise on the Cultivation of the Voice, with a Discussion of Principles and Suggestions for Practice. By George B. Hynson, M. A. 415 pages, illustrated. Price, $3.50. Detriot: George Andrew Lewis. 1902.

Mr. Lewis takes the stand that in the vast majority of cases these disorders are of nervous origin without appreciable organic lesions and as such are treatable more by elocutionary methods than by operative, as was formerly thought. The work is devoted to an elaboration of this view and many practical and helpful suggestions are given, including prose and poetical selections especially adapted to the author's methods.

We recommend the work to all having an interest in the subject. In part II, on the cultivation of the voice, Mr. Hynson laments the ill use we so frequently make of that priceless gift, and makes a plea for its better cultivation during youth.

In offering this work to the profession, the author very aptly makes the point that the physician and surgeon are frequently called in by teachers and parents relative to vocal troubles in children, and so should know the possibilities of voice treatment by elocutionary means. We most heartily agree with this idea, and take pleasure in recommending the work. J. M. L.

